# Disability and HIV: What drives this relationship in Eastern and Southern Africa?

**DOI:** 10.4102/ajod.v2i1.25

**Published:** 2013-02-12

**Authors:** Jill Hanass-Hancock, Ilaria Regondi, Kerisha Naidoo

**Affiliations:** 1 Health Economics and HIV and AIDS Research Division (HEARD), University of KwaZulu-Natal, South Africa

## Abstract

The Eastern and Southern Africa (ESA) region is the epicentre of the global HIV epidemic and also home to a large number of people with disabilities. Both HIV and Disability are significant public health issues. While they are generally viewed as distinct and unrelated phenomena data seems to suggest that they are particularly closely intertwined in ESA.

For the first time in history, by using the same disability indicator consistently, the publication of the World Report on Disability in 2011 has allowed for the comparison of disability data between countries, and across regions. This has the potential to shed some light on the relationship between disability and socio-economic markers and other health conditions in a way that was not possible previously. In the absence of disability and HIV-specific population-based surveys, this paper uses global socio-economic and HIV datasets and compares them to data contained in the most recent World Report on Disability.

The analysis suggests that disability prevalence may be related to HIV-prevalence in ESA (Pearson 0.87). It identifies research and policy gaps and seeks to shed light on the relationship between the two phenomena. It concludes that, more than any other region in the world, ESA needs to ensure better data collection on disability and the inclusion of disability throughout its HIV programmes in order to provide a comprehensive and appropriate response to the epidemic.

## Introduction

Disability and HIV might be more interrelated in Eastern and Southern Africa (ESA) than in any other part of the world. It is well known that the ESA region is the epicentre of the global HIV epidemic (Joint United Nations Programme on HIV/AIDS [UNAIDS] 2010; UNAIDS 2011). Approximately 68% of all people living with HIV (PLHIV) reside in sub-Saharan Africa, which includes ESA (UNAIDS 2011). What may be less well known is that the latter is also home to very high disability prevalence rates; however, until recently it was impossible to compare disability data across countries as the use of indicators and data collection varied too greatly.

In 2011, the World Report on Disability was released by the World Health Organisation (WHO) and the World Bank. In using the same indicator across countries, for the first time in history, the report provided the global community with valuable and comparable disability prevalence data. This indicator uses a comprehensive definition and measure of disability, which captures activity limitations and their intensity, rather than merely capturing a more overt concept of impairment (which is based on the medical concept of disability).

The report states that the International Classification of Functioning, Disability and Health (ICF) was used as a conceptual framework to guide this approach (World Health Organization & The World Bank 2011). The benefit of this approach cannot be overemphasised. Previously, disability measures differed from country to country, thus making cross-country comparisons very difficult. This paper takes advantage of newly-available data to analyse global and regional HIV, disability and socio-economic data in a way never before possible. The paper will focus on the ESA region, which has the highest HIV-prevalence in the world.

Using the WHO and/or World Bank disability indicator, the World Report on Disability suggests that disability prevalence in the ESA region is between 14% and 36%, including different types and degrees of disability. Swaziland has the highest disability prevalence (35.9%) and South Africa, which hosts more people living with HIV than any other country in the world, has a disability prevalence of over 24% (WHO/World Bank 2011). Therefore, people with disability constitute a significant proportion of ESA, yet HIV programming in the region is not accessible to and inclusive of people with disabilities, nor does it provide for HIV-related disability, as argued by much of the relevant literature (Groce 2004; Hanass-Hancock 2009; Nixon *et al*. 2011b; Rohleder & Swartz 2009; Swartz, Schneider & Rohleder 2006; UNAIDS 2009; Wazakili 2010).

In the mid-2000s, the roll-out of anti-retroviral treatment (ART) in ESA increased the life expectancy and survival rate of people infected with HIV (UNAIDS 2010; UNAIDS 2011). However, along with this progress, the risk of experiencing HIV-related disabilities also increased (Hanass-Hancock & Nixon 2009; Myezwa *et al*. 2011; Nixon *et al*. 2011a; Nixon *et al*. 2011b). This research area has received some attention in resource-rich settings, where ART has been available since the mid-1990s (Hanass-Hancock & Nixon 2009). However, it remains relatively unexplored in resource-poor settings, such as ESA. Similarly, while some data is available on the relationship between mental health and HIV (Brandt 2009), limited research has been conducted on the connection between HIV and other kinds of disabilities in Africa (Brandt 2009; Hanass-Hancock 2009; Smart 2009). Some literature focuses on HIV-related impairments in the region, such as neurocognitive impairments, HIV dementia, neuropathy, epilepsy, hypertension, and motor delays in children (Burton 2010; Del Rio, Foyaca-Sibat & Ibanez-Valdez 2007; Ferguson & Jelsma 2009; Joseph & Prasad 2005; Joska *et al*. 2010; Lawler *et al*. 2010; Lawler *et al*. 2011; Lowe *et al*. 2010; Maritz *et al*. 2010; Wong *et al*. 2007; Yengopal & Naidoo 2008), yet seldom have these conditions been connected to the broader concepts of disability and rehabilitation (Brandt 2009; Hanass-Hancock & Grant 2010; Myezwa *et al*. 2009; Nixon *et al*. 2011b). It is therefore not surprising that national programmes on HIV and National Strategic Plans (NSPs) seldom include the concept of disability, with many failing to address HIV-related disability altogether (Hanass-Hancock & Grant 2010).

This paper highlights some contemporary thinking about HIV and disability, and in so doing, begins to explore this relationship. It discusses some of the commonly-described key factors driving disability and poses a number of questions: to what extent are HIV and disability interrelated, and can HIV be one of the driving factors behind high disability rates in the region? The paper uses the scattered literature on disability and HIV as well as publicly available international datasets in order to explore trends and relationships between disability and a number of socio-economic variables. Disability prevalence, as mentioned above, was extracted from the 2011 World Report on Disability; HIV-prevalence was extracted from the 2011 UNAIDS report, and the socio-economic datasets from the World Development Indicators database (World Bank n.d.) and the 2011 Human Development Report (United Nations Development Programme 2011). It is hoped that some of the ideas that are raised in this exploratory paper will help spearhead further research on the link between disability and HIV.

## Factors driving disability

Disability can be understood on three different levels; namely impairment, activity limitation, and participation restriction levels. Whilst impairment is often a result of acquired health conditions, the other two levels are a result of ‘inaccessible environments that cause disability by creating barriers to participation and inclusion’ (WHO/World Bank 2011).

The World Report on Disability highlights several risk factors that drive impairment and/or disability. These factors include infectious diseases (HIV, tuberculosis [TB] and sexually-transmitted [STIs]); non-communicable chronic diseases (such as diabetes and cancer); injuries (including road traffic accidents, violence and occupational injuries); environmental conditions (poor sanitation, poverty, natural disasters and conflict situations); and old age, as the chances of becoming disabled increase with age (WHO/World Bank 2011). The literature on disability places significant emphasis on disease, injuries, poverty, and old age (Banda 2005; Braithwaite & Mont 2009; CASS Centre for Approved Social Science & Rekopantswe 2007; Choruma 2006; Elwan 1999; Emmett 2006; Handicap International 2011a; Mitra, Posarac & Brandon 2012; Watermeyer *et al*. 2006) and their contribution to the development of disability. For instance, the link between poverty and disability is often discussed as a ‘vicious circle’ (Handicap International 2011a; Mitra *et al*. 2012), where poverty features as one of the key drivers of disability; disability may in turn lead to impoverishment due to lack of opportunities and access to health services, education, employment, et cetera (Elwan 1999; Emmett 2006; Mitra *et al*. 2012; WHO/World Bank 2011).

Similar to Gillespie’s analysis of HIV and economics (Gillespie *et al*. 2007) it is interesting to explore how disability relates to key socio-economic indicators such as Gross National Income (GNI), the GINI coefficient, a common measure of income inequality, and the Human Development Index (HDI), a composite index and comparative measure of life expectancy, literacy, education and standards of living. Intuitively, a negative relationship between disability and these socio-economic indicators could be expected, but, using data from both developed and developing countries, this paper found that on a global level, there was only a very weak correlation between disability on the one hand and HDI, GINI and GNI on the other.

However, one could argue that in different regions of the world, disability is driven by different factors and therefore the correlation is weak or non-existent. For instance, in low-income countries disability is likely to be driven by malnutrition, conflict, and poverty, while in more developed countries it could be driven by an aging population and an increase in non-communicable diseases. A region- or country-specific analysis might, however, be more appropriate. The analysis for this paper was, therefore, an ESA-specific analysis, with the intention of exploring how the linkages between disability and HIV, as well as other socio-demographic factors, play out in a high HIV-prevalence area such as ESA. A limitation of the analysis is that only data from those countries where both disability and HIV data was available were used. While the 2011 UNAIDS report included all ESA countries except Ethiopia, the World Disability Report only included a selection of countries. Not all countries were selected for the WHO survey, so a common and comparable disability indicator is only available in the selected countries. The World Disability Report states that the countries selected for the global survey were chosen using a stratified, multistage cluster (WHO 2011). This paper used the WHO dataset and compared it to other datasets as described above for the ESA region (see Table 1).

**TABLE 1:** Socio-economic, disability and HIV data for Eastern and Southern Africa.

Countries	Disability prevalence, % (2010/2011)	HIV-prevalence, % (2009/2010)	GNI per capita in PPP, intl $ (2005)	HDI value (2011)
Kenya	15.2	6.3	1.492	0.51
Malawi	14	11	753	0.40
Mauritius	13.1	1	12.918	0.73
Namibia	21.4	13.1	6.206	0.63
South Africa	24.2	17.8	9.469	0.62
Swaziland	35.9	25.9	4.484	0.52
Zambia	14.8	13.5	1.254	0.43
Zimbabwe	16.9	14.3	376	0.38

*Source*: World Bank n.d.; WHO & The World Bank 2011 GNI, Gross National Income; PPP, purchasing poert parity; intl, international; HDI, Human Development Index.

**FIGURE 1:**
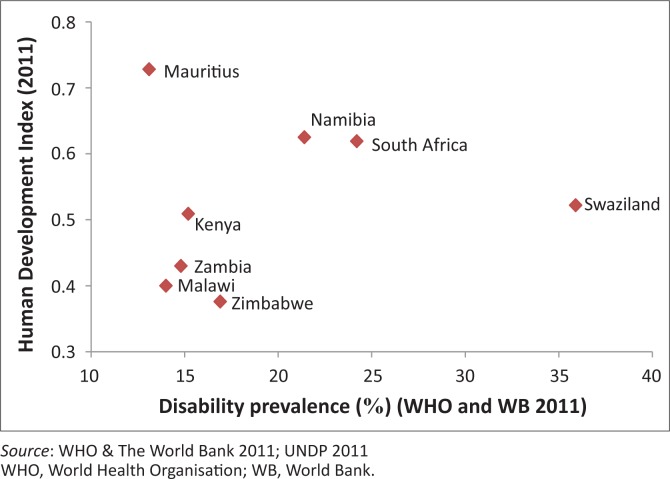
Disability and Human Development Index in Eastern and Southern Africa.

**FIGURE 2:**
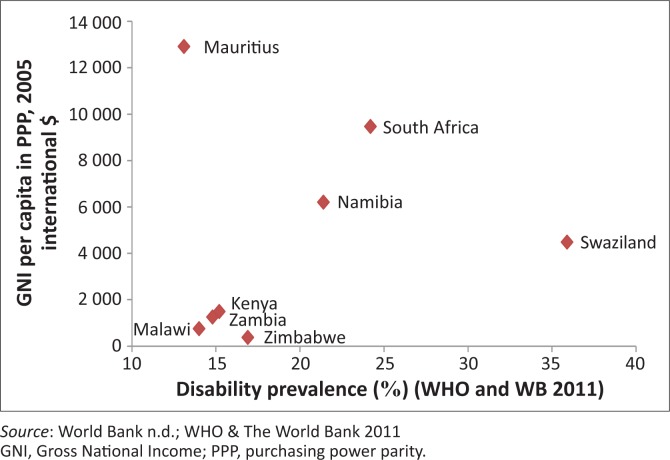
Disability and Gross National Income in Eastern and Southern Africa.

Our analysis of these datasets found no strong association between disability and HDI, GINI or GNI, as illustrated in Figures 1 and 2. These findings may suggest that even in this region, disability may be driven by numerous factors, and that its relationship with poverty, education, health, and so on is a complex one and more difficult to highlight as in the case of HIV. Whilst academics in the field of economics have established a link between increased HIV-prevalence and inequality using the same data (Gillespie *et al*. 2007), Figures 1 and 2 illustrate that the same connection cannot be shown between disability and inequality or poverty indicators (GINI, HDI or GDP)^1^. A few of the higher-income countries of the region – such as South Africa, Namibia and Swaziland – actually exhibit very high disability prevalence rates, thus running contrary to common assumptions about the positive relationship between disability and poverty. If poverty, education and standards of living are not clearly driving disability in this region, what else could be at play?

**FIGURE 3:**
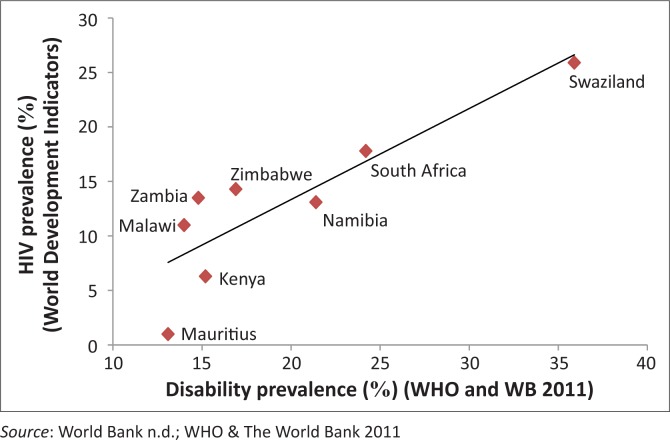
Disability and HIV-prevalence.

Health and access to health services is one of the other factors and plausible links to disability (WHO/World Bank 2011). As ESA is a high HIV- and TB-prevalence area, one could argue that disability might be driven in part by these diseases and their treatment (Meintjes *et al*. 2012; Nixon *et al*. 2011b). An investigation ensued to determine whether HIV could indeed be a key factor associated with disability in this region. To explore the feasibility of this argument, the prevalence of disability and HIV globally, in both developing and developed countries, was correlated. Interestingly, no such association was found on a global scale. However, as Figure 1 shows, when restricting the analysis to countries in the ESA region specifically, a strong correlation (*r* = 0.87) was found. Even when controlling for outliers, the correlation still yielded a high Pearson’s value of 0.71 (Figure 3). This shows nothing more than that the countries in ESA that are burdened with high HIV-prevalence are also those with high disability prevalence. Whilst correlation certainly cannot be equated with causation, this finding does provide some scope for reflection.

As there is currently a paucity of research on both HIV-related disability and on people with both disability and HIV, exploration of the link between the two phenomena is needed urgently. Given the millions of people who are infected with HIV in the region, this relationship could have massive health, social and economic implications. In the era of ART, with all its side effects and potentially disabling associated conditions (Meintjes *et al*. 2012; Nixon *et al*. 2011b), this realisation is particularly important. However, because of the lack of population-based data, we can only speculate about the relationship between disability and HIV; there is little information about both the extent of HIV-related disability in the region and HIV-prevalence rates among people with disabilities. The few studies that exist indicate diverse adverse effects to all available ART drugs (Meintjes *et al*. 2012), possible high prevalence of HIV-related disability including those people on ART (Myezwa *et al*. 2011), as well as high HIV-prevalence amongst people with disabilities (Shisana *et al*. 2009), yet these are only isolated study results, mainly focusing on South Africa.

Without population-based knowledge, it is difficult for both policy-makers and practitioners, in both the public and voluntary sector, to plan accurately and provide appropriate services on the right scale.

## Disability and HIV: Pathways and interactions

While the need for a deeper understanding has been established, and although it is difficult to identify clear pathways and interactions between disability and HIV, the link between the two remains a largely under-researched field. However, the available data suggests that people with disabilities are at increased risk of exposure to HIV (Groce 2004; Hanass-Hancock 2009; UNAIDS 2009) and that PLHIV are at risk of developing impairments that can lead to disability as a result of their illness (including opportunistic infections) or their treatment, given the toxicity and associated adverse reactions and sometimes poor absorption of ARTs (Brandt 2009; Meintjes *et al*. 2012; Myezwa *et al*. 2009; Myezwa *et al*. 2011; Nixon *et al*. 2011a; Nixon *et al*. 2011b; Sherr *et al*. 2011; Smart 2009).

On the one hand, people with disabilities are seen as an at-risk population because they are exposed to well-known HIV risk factors such as poverty, inadequate sex education, poor access to health services, risk of sexual abuse, and partner fluctuation, to which women and girls with disabilities are particularly vulnerable (Banda 2005; Groce 2004; Hanass-Hancock 2009; Rohleder & Swartz 2009; Swartz *et al*. 2006; Touko et al. 2010; UNAIDS 2009; Watermeyer *et al*. 2006; Wazakili 2010). The few studies that measure HIV-prevalence amongst people with disabilities in Africa support these arguments by revealing similar or higher HIV-prevalence rates amongst people with disabilities than their able-bodied peers (Shisana *et al*. 2009; Taegtmeyer *et al*. 2009; Touko *et al*. 2010). On the other hand, studies are emerging which provide some insight into the extent (in terms of numbers and conditions) to which disability is an increasingly common problem in relation to HIV and its treatment (Meintjes *et al*. 2012; Nixon *et al*. 2011b).

## Existing literature and scope for further research

A number of medical studies which focus predominantly on the impairment level identify several HIV-related impairments. These include HIV dementia, neurocognitive disorders, peripheral neuropathy, blindness, skin problems, fatigue, strokes, depression and many others (Maritz *et al*. 2010; McGrath & Cooke 2007). Activity limitations are often identified in studies that use Quality of Life Scales in exploring the impact of HIV as a chronic illness (Mannheimer *et al*. 2005; McInerney *et al*. 2008; Pate *et al*. 2009). They point to issues with mobility and household activities. The extensive literature that exists on the stigma and discrimination that surround HIV relates to participation restrictions. Helpfully, all of the studies that use the ICF model and its related tools cover all three aspects of disability, as identified by the framework itself.

An example of one such study is by Myezwa *et al*. (2011), who compared four different studies that used the ICF framework in resource-poor settings. The paper illustrates that PLHIV experience pain, cardiovascular function disorders, digestive function problems, especially weight maintenance, decreased sexual and reproductive functions, loss of muscular power, and skin problems. Although the sample size in each of these studies was limited, the extent of HIV-disability was clearly not. Mental functions presented the most problems, with sleep, energy and drive, and emotional functions being the most affected. In those who were undertaking long-term therapy, body image was a key issue for the majority of people surveyed. Decreased mobility, ability to self-care and perform domestic tasks, as well as ability to remain at work were other commonly cited problems, which could eventually lead to disability.

Unfortunately, this paper has only been able to raise questions rather than provide definitive answers, given that there is no population-based data available for the ESA region. There is a lack of such data for both HIV-related disability and people with disability and HIV. Most available studies are conducted on a small scale and provide insight into the relationship between disability and HIV, but do not provide enough information about how the link plays out on a population level. As mentioned earlier, this data is crucial to inform HIV programmes in the region.

On the one hand, people with disabilities have the right to access HIV prevention, treatment and care. Using a human rights perspective, the need to focus on intervention research in this field can be argued. However, in order to provide the right disability data, there is also a need to advocate for the inclusion of disability-related questions in larger household surveys, HIV-prevalence studies or intervention studies. Furthermore, there is little information available on HIV-related disability which focuses specifically on resource-poor settings. The urgency to better understand the relationship in more depth was highlighted in special sessions at the disability networking zone, the rapporteur session at the ICASA conference in Ethiopia in December 2011, as well as at the XIX International AIDS conference in Washington 2012 (Hanass-Hancock, Mac-Seing & Timpo 2012; Handicap International 2011b; HEARD 2011). This provides hope that the issue may gain greater prominence in the operations, actions and funding strategies of the many actors and stakeholders present in the field.

## Policy Implications

From a policy perspective, HIV programming will increasingly have to address and include disability in its response to prevention, treatment, care and support. Thus far, progress has been limited. A 2010 systematic review of all National Strategic Programmes (NSPs) on HIV in the ESA region revealed that only a few countries identified disability as an issue in their response to HIV, and that none of them addressed HIV-related disability (Hanass-Hancock, Strode & Grant 2011). At the same time, many ESA countries have signed the UN Convention on the Rights of Persons with Disability and are therefore obliged to address disability. Additionally, many NSPs are currently under review, and this provides an invaluable opportunity to positively influence the shape and content of future plans. The recently-launched disability-inclusive NSP framework provides valuable guidelines and tools on how best to develop these, and at the same time fulfil countries’ obligations under the UN Convention (NSP Task Group on Disability & HIV 2011). This framework was launched by UNAIDS, Handicap International and HEARD at ICASA (Handicap International 2011b; HEARD 2011) and taken up in a skills building workshop at the XIX International AIDS Conference 2012 (Hanass-Hancock *et al*. 2012). The workshop was highlighted in the final conference rapporteur session:

We learnt also from the skills-building workshop on the inclusion of disability in national strategic plans that despite the ratification of the CRPD many countries have not addressed the issues of this group which accounts for 15% of the world’s population … We were told to tell you that universal access, zero infections, zero AIDS-related death and zero discrimination cannot be achieved without including the world’s largest minority: the disabled. (Volderine Hacket, Leadership and Accountability Programme Rapporteur Report, XIX International AIDS Conference, Washington, 2012)

To sum up, particularly in ESA, responses to HIV and AIDS can no longer feasibly be conceived separately from responses to disability.

## Conclusion

The picture painted above as well as a number of reviews in the field (Brandt 2009; Hanass-Hancock 2009; Meintjes *et al*. 2012; Rohleder *et al*. 2009) provide a glimpse into the possible ways in which HIV and disability may be related. The link between these two phenomena might be greater in high HIV-prevalence countries than elsewhere, where wars, accidents, poverty, age, diseases and other factors could be driving disability more dominantly. The sparse literature that is currently available on disability and HIV provides little information and is neither conclusive nor exhaustive. Further research is urgently needed in order to prepare the region for the current and future impact of both issues. From a practical perspective, HIV programmes need to integrate disability into their activities in a more effective manner in order to offer comprehensive responses to the epidemic and the impairments, restrictions and limitations that it may bring. NSPs might be a key document to work with at this point. The consequences of not doing so in ESA, given the extent of the epidemic, would not only be a human rights disaster, but will also have an impact on many other issues in the region such as socio-economic issues, as people survive but with less ability to support their livelihoods if rehabilitation is not available. Therefore, the urgency of this task, from a health, social and economic point of view, cannot be overstated.

## Footnotes

1 Figures 1 and 2 portray the association between Disability prevalence figures from the World Bank and WHO, compared with HDI figures obtained from the UNDP (Figure 1) and GNI in ESA (Figure 2). The figures show no association between disability and socio-economic indicators.
